# Supplementary material for: Measuring changes in transmission of neglected tropical diseases, malaria, and enteric pathogens from quantitative antibody levels
Source: PLoS Negl Trop Dis. 2017 May 19;11(5):e0005616. doi: 10.1371/journal.pntd.0005616 (PMC5453600; doi:10.1371/journal.pntd.0005616)
Supplement: S1 Text — (PDF) [file pntd.0005616.s001.pdf]

Measuring changes in transmission of neglected tropical diseases, malaria, and enteric pathogens from quantitative antibody levels

S1 Text: Relationship between the age-adjusted mean antibody response and the area under the curve

As in the main text Methods, the observed data on individuals include a quantitative antibody response (Y), age (A), a categorical exposure of interest (X), and a set of potentially confounding covariates (W). We observe n i.i.d. copies of $O = (Y, A, X, W)$ with probability distribution $O \sim P_0$. Age-antibody curves are the mean antibody response by age ($A = a$) and exposure ($X = x$), marginally averaged over W:

$$E(Y_{a,x}) = E_W\{E(Y|X = x, A = a, W)\} \qquad (1)$$

A smooth function of the age-antibody curve is the overall mean antibody level conditional on exposure group ($X = x$):

$$E(Y_x) = E_{A,W}\{E(Y|X = x, A, W)\} \qquad (2)$$

The following illustrates the relationship between this marginal mean and the area under the age-antibody curve:

$$\begin{aligned}
E(Y_x) &= \int_w \int_a E(Y|X = x, A = a, W = w)P(A = a|W = w)P(W = w) \\
&= \int_w \int_a E(Y_{a,x}|W = w)P(A = a|W = w)P(W = w) \\
&= \int_w \left[\int_a E(Y_{a,x}|W = w)P(A = a|W = w)\right] P(W = w) \\
&= E_W \left[\int_a E(Y_{a,x}|W = w)P(A = a|W = w)\right]
\end{aligned}$$

The term inside the brackets is area under the age-antibody curve (AUC), within strata defined by $W = w$ and weighted by $P(A = a|W = w)$. $E(Y_x)$ is thus the

marginal average across the straum-specific AUCs. In a special case where age is independent of other covariates, or investigators do not need to condition on potential confounders, $P(A = a|W) = P(A = a)$, and the above expression further reduces to:

$$E(Y_x) = \int_a E(Y_{a,x}) P(A = a) \tag{3}$$

This quantity is the AUC of the age-antibody curve in exposure group $X = x$.
