# Supplementary material for: Measuring changes in transmission of neglected tropical diseases, malaria, and enteric pathogens from quantitative antibody levels
Source: PLoS Negl Trop Dis. 2017 May 19;11(5):e0005616. doi: 10.1371/journal.pntd.0005616 (PMC5453600; doi:10.1371/journal.pntd.0005616)
Supplement: S2 Text — (PDF) [file pntd.0005616.s002.pdf]

Measuring changes in transmission of neglected tropical diseases, malaria, and enteric pathogens from quantitative antibody levels

S2 Text: Technical details of estimating age-dependent antibody curves and changes in mean antibody levels

Targeted maximum likelihood estimation (TMLE) is a double-robust, efficient estimation approach that targets the fit of the likelihood to the target parameter of interest [1, 2]. Below, we describe details of the estimation process and link the statistical estimation procedure directly to the observed data through a causal model [3]. TMLE is implemented in R using the `tmle` package (see [4] for a helpful overview of the package and methodology). A full set of replication files to implement the analyses described in this article, including a companion R package (`tmleAb`) and vignette, are available through GitHub and the Open Science Framework: `https://osf.io/8tqu4`.

Observed data and causal model

As in the main text Methods, a cross-sectional survey measures an individual's quantitative antibody level (Y), age (A), and other characteristics (W). Many surveillance efforts are also interested in differences in antibody levels by one or more exposures (X), which may be confounded by W. We assume the observed data $O = (Y, A, W, X) \sim P_0$ arise from a simple causal model:

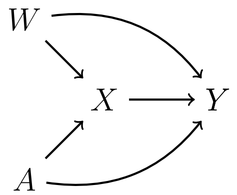

$$\begin{aligned} W &= f_W(U_W) \\ A &= f_A(U_A) \\ X &= f_X(A, W, U_X) \\ Y &= f_Y(X, A, W, U_Y) \end{aligned}$$

For simplicity, the graph on the left omits unmeasured characteristics (U) that,

together with each variable's parents, determine what value it takes (e.g., the error term for X is denoted U_X). The equations encode assumptions about time-ordering between variables in the data (e.g., X, A, and W precede Y), but make no assumptions about the functional form of the relationship between them. The equations make no formal assumption about the relationship between errors that generate the data, but under the untestable assumption of no unmeasured common causes of Y and X, (independence of U_Y and U_X), then conditional on A and W mean differences between groups defined by X have a causal interpretation.

Fitting age-dependent antibody response curves

We are interested in a nonparametric model the mean antibody response as a function of age A, exposure X and potential additional covariates W (all defined above).

$$E(Y_{a,x}) = E_W[E(Y|A = a, X = x, W)], \tag{1}$$

where the outside expectation marginally averages over the covariates W. A flexible approach to modeling the mean is to use an ensemble of models and algorithms to predict the mean over observed values of age and exposure. To accomplish this, we used the "super learner" algorithm, which is a stacked regression approach that combines individual predictions from each model or algorithm in its library into a single prediction that minimizes the cross-validated loss – in this case the mean squared error [5]. The algorithm is implemented in the `SuperLearner` package in R, and the companion R package for this paper (`tmleAb`) includes a convenient interface for flexibly estimating antibody curves.

In this analysis, we included in the ensemble: the simple mean, generalized linear models, antibody acquisition models with constant rates [6], locally weighted regression (lowess) [7], generalized additive models with natural splines [8], multivariate adaptive regression splines [9], and Random Forest [10]. We used default tuning parameters with two exceptions. First, for generalized additive models we selected the degrees of freedom smoothing parameter in natural splines for each fit using cross-validation [11]. Second, the default Random Forest implementation in R can overfit

the data by growing trees that are too deep (i.e., have too small nodes), so for each fit we selected the minimum node size in Random Forest using cross-validation.

After fitting the ensemble, we generated curves by predicting the mean outcome for each level of age and exposure each analysis. Nonparametric estimates of an exposure-response curve with a continuous exposure (here: age) do not converge at a $n^{1/2}$ rate [12] so pointwise confidence intervals will typically have poor coverage, even under a semi-parametric generalized additive model [13]. Below, we propose a summary function of the curve, the mean averaged over a range of ages, which can be estimated consistently and efficiently under a nonparametric model.

Target parameter of interest (estimand) to compare groups

Our parameter of interest of the observed data distribution P_0 was the difference in the age- and covariate-adjusted population average antibody response in different groups. For example, in the Mauke study the exposed group ($X = 1$) included measurements after mass drug administration (MDA), and unexposed ($X = 0$) was pre-MDA:

$$\Psi(P_0) = \psi_0 = E_{P_0}\left[E_{P_0}(Y|X=1,A,W) - E_{P_0}(Y|X=0,A,W)\right], \tag{2}$$

where the outer expectation is the mean averaged over the empirical distribution of A and W. Ψ represents a mapping from a probability distribution P_0 to a real number – namely, the difference in means. This target parameter depends on the probability distribution P_0 of O through two quantities: the conditional mean, $E_{P_0}(Y|X,A,W)$, and the marginal distribution of (A,W). Due to the high dimensionality of the statistical model, standard maximum likelihood estimation, which maximizes the likelihood over all possible probability distributions, is not possible or results in overfitted estimators. Instead, targeted maximum likelihood (TMLE) is a two stage procedure that first uses ensemble machine learning to obtain an initial fit of these quantities and subsequently targets the fit so that it is optimal for the resulting plug-in estimator of ψ_0.

Targeted maximum likelihood estimation (TMLE)

For n observations from the probability distribution $O \sim P_0$, we define a plug-in estimator of ψ_0 with the form:

$$\hat{\psi} = \frac{1}{n}\sum_{i=1}^{n}\left[\hat{E}^*(Y_i|X_i = 1, A_i, W_i) - \hat{E}^*(Y_i|X_i = 0, A_i, W_i)\right]. \tag{3}$$

TMLE estimates $\hat{\psi}$ using a two-stage process. The first stage makes an initial estimate of $\hat{E}^*(Y|X, A, W)$, denoted $\hat{E}^0(Y|X, A, W)$. This could be done using a highly parametric approach, such as linear regression, but the relationship between antibody response, exposure group, age, and other characteristics could be very complex. As with the estimation of age-dependent antibody curves, we used the "super learner" algorithm to flexibly estimate $\hat{E}^0(Y|X, A, W)$, which are predicted antibody levels conditional on X, A, and W.

The second stage of TMLE updates the the initial fit, $\hat{E}^0(Y|X, A, W)$, using a targeting step. If the initial estimate of $\hat{E}^0(Y|X, A, W)$ is consistent then the TMLE estimate is consistent, but if it is biased then the updating step helps remove residual bias in the estimation of ψ_0. It involves first estimating a nuisance parameter: the probability of treatment given observed characteristics, sometimes called the propensity score [14]. In randomized trials $P(X = x|A, W)$ is known, but in observational studies $P(X = x|A, W)$ is typically not known and must be estimated. We estimated $\hat{P}(X = x|A, W)$ using the super learner algorithm with the same library of learners described above, but with X as a binary outcome variable. The analysis assumes that $P_0(X = 1|A = a, W = w) > 0$ and $P_0(X = 0|A = a, W = w) > 0$ are positive. Without this assumption, the conditional expectations of Y in $\Psi(P_0)$ are not well defined. In practice, this second assumption means that exposure groups need to have good overlap in age and any other covariates included in the analysis.

TMLE then uses $\hat{P}(X = x|A, W)$ to construct an individual-level covariate, which is chosen specifically for our parameter of interest (equation 2) to solve the efficient

influence curve and thus minimize the bias in the estimate of ψ_0 [2]:

$$h(X, A, W) = \left(\frac{I(X = 1)}{\hat{P}(X = 1|A, W)} - \frac{I(X = 0)}{\hat{P}(X = 0|A, W)} \right) \tag{4}$$

The targeted update is achieved using a univariate generalized linear model that includes $\hat{E}^0(Y|X, A, W)$ as the offset with $h(X, A, W)$ as a single covariate. For continuous outcomes such as Y, it has been shown that a conducting the update on the linear scale works in many cases (i.e., a generalized linear model with an identity link), but if $h(X, A, W)$ can take on large values then there are important robustness advantages to conducting the update on the logistic scale using a re-scaled version of the outcome bound between (0,1) [15]. If Y is bounded by (a, b) then a re-scaled version $Y^* = (Y - a)/(b - a)$ is bounded by (0,1). The logistic model is then fit using maximum likelihood:

$$\text{logit}[\hat{E}^1(Y^*|X, A, W)] = \text{logit}[\hat{E}^0(Y^*|X, A, W)] + \hat{\epsilon} h(X, A, W) \tag{5}$$

Predicted antibody levels from this update, transformed back to their original scale, are then used in the plug-in estimator: $\hat{E}^1(Y|X, A, W) = \hat{E}^*(Y|X, A, W)$ in equation 3. TMLE is a regular, asymptotically linear estimator. For this reason, standard errors, confidence intervals, and P-values are estimated using the influence curve [1, 2], and readily accommodate repeated measures data if that is a feature of the design [4].

References

[1] van der Laan MJ, Rubin D. Targeted Maximum Likelihood Learning. Int J Biostat. 2006;2(1):1–38.

[2] van der Laan MJ, Rose S. Targeted Learning: Causal Inference for Observational and Experimental Data. Springer Series in Statistics; 2011.

[3] Petersen ML, van der Laan MJ. Causal models and learning from data: integrating causal modeling and statistical estimation. Epidemiology. 2014 May;25(3):418–426.

[4] Gruber S, van der Laan M. tmle: An R Package for Targeted Maximum Likelihood Estimation. J Stat Softw. 2012 16 Nov;51(13):1–35.

[5] van der Laan MJ, Polley EC, Hubbard AE. Super Learner. Stat Appl Genet Mol Biol. 2007;6(1):1544–6115.

[6] Yman V, White MT, Rono J, Arcà B, Osier FH, Troye-Blomberg M, et al. Antibody acquisition models: A new tool for serological surveillance of malaria transmission intensity. Sci Rep. 2016 5 Feb;6:19472.

[7] Cleveland WS, Devlin SJ. Locally weighted regression: an approach to regression analysis by local fitting. J Am Stat Assoc. 1988;83(403):596–610.

[8] Hastie T, Tibshirani R. Generalized Additive Models. London: Chapman and Hall; 1990.

[9] Friedman JH. Multivariate Adaptive Regression Splines. Ann Stat. 1991;19(1):1–67.

[10] Breiman L. Random Forests. Mach Learn. 2001;45(1):5–32.

[11] Hastie T, Tibshirani R, Friedman J. The Elements of Statistical Learning. 2nd ed. New York: Springer; 2009.

[12] Bickel PJ, Klaassen CAJ, Ritov Y, Wellner JA, Others. Efficient and adaptive estimation for semiparametric models. 1998;.

[13] Marra G, Wood SN. Coverage Properties of Confidence Intervals for Generalized Additive Model Components. Scand Stat Theory Appl. 2012 1 Mar;39(1):53–74.

[14] Rosenbaum PR, Rubin DB. The central role of the propensity score in observational studies for causal effects. Biometrika. 1983;70(1):41–55.

[15] Gruber S, van der Laan MJ. A targeted maximum likelihood estimator of a causal effect on a bounded continuous outcome. Int J Biostat. 2010 1 Aug;6(1):Article 26.
