# Supplementary material for: Measuring changes in transmission of neglected tropical diseases, malaria, and enteric pathogens from quantitative antibody levels
Source: PLoS Negl Trop Dis. 2017 May 19;11(5):e0005616. doi: 10.1371/journal.pntd.0005616 (PMC5453600; doi:10.1371/journal.pntd.0005616)
Supplement: S1 Table — (PDF) [file pntd.0005616.s003.pdf]

Measuring changes in transmission of neglected tropical diseases, malaria, and enteric pathogens from quantitative antibody levels

S1 Table. Strengthening the Reporting of Observational Studies in Epidemiology (STROBE) Checklist

Item	DESCRIPTION	REPORTED IN SECTION
**Title and Abstract**		
1a	Indicate the study's design with a commonly used term in the title or the abstract	Abstract (repeated cross-sectional surveys)
1b	Provide in the abstract an informative and balanced summary of what was done and what was found	Abstract
**Introduction**		
Background/rationale		
2	Explain the scientific background and rationale for the investigation being reported	Introduction
Objectives		
3	State specific objectives, including any prespecified hypotheses	Introduction
**Methods**		
Study Design		
4	Present key elements of study design early in the paper	Methods -<br>- Overview of the approach
Setting		
5	Describe the setting, locations, and relevant dates, including periods of recruitment, exposure, follow-up, and data collection	Methods -<br>- Lymphatic filariasis transmission on Mauke Island<br>- Malaria transmission in the Garki Project, Nigeria<br>- Enteric pathogen transmission in Haiti and the United States
Participants		
6a	Give the eligibility criteria, and the sources and methods of selection of participants. Describe methods of follow-up	Methods -<br>- Lymphatic filariasis transmission on Mauke Island<br>- Malaria transmission in the Garki Project, Nigeria<br>- Enteric pathogen transmission in Haiti and the United States
6b	For matched studies, give matching criteria and number of exposed and unexposed.	NA

<table>
<tr><td colspan="3">Variables</td></tr>
<tr><td>7</td><td>Clearly define all outcomes, exposures, predictors, potential confounders, and effect modifiers. Give diagnostic criteria, if applicable</td><td>Methods -
- Lymphatic filariasis transmission on Mauke Island
- Malaria transmission in the Garki Project, Nigeria
- Enteric pathogen transmission in Haiti and the United States</td></tr>
<tr><td colspan="3">Data Sources and Management</td></tr>
<tr><td>8</td><td>For each variable of interest, give sources of data and details of methods of assessment (measurement). Describe comparability of assessment methods if there is more than one group</td><td>Methods -
- Lymphatic filariasis transmission on Mauke Island
- Malaria transmission in the Garki Project, Nigeria
- Enteric pathogen transmission in Haiti and the United States</td></tr>
<tr><td colspan="3">Bias</td></tr>
<tr><td>9</td><td>Describe any efforts to address potential sources of bias</td><td>Methods
- Statistical methods</td></tr>
<tr><td colspan="3">Study Size</td></tr>
<tr><td>10</td><td>Explain how the study size was arrived at</td><td>NA</td></tr>
<tr><td colspan="3">Quantitative Variables</td></tr>
<tr><td>11</td><td>Explain how quantitative variables were handled in the analyses. If applicable, describe which groupings were chosen and why</td><td>Methods
- Statistical methods</td></tr>
<tr><td colspan="3">Statistical Methods</td></tr>
<tr><td>12a</td><td>Describe all statistical methods, including those used to control for confounding</td><td>Methods
- Statistical methods
- S1 Text
- S2 Text</td></tr>
<tr><td>12b</td><td>Describe any methods used to examine subgroups and interactions</td><td>NA</td></tr>
<tr><td>12c</td><td>Explain how missing data were addressed</td><td>NA</td></tr>
<tr><td>12d</td><td>If applicable, explain how loss to follow-up was addressed</td><td>NA</td></tr>
<tr><td>12e</td><td>Describe any sensitivity analyses</td><td>None</td></tr>
<tr><td colspan="3">Results</td></tr>
<tr><td colspan="3">Participants</td></tr>
<tr><td>13a</td><td>Report numbers of individuals at each stage of study—eg numbers potentially eligible, examined for eligibility, confirmed eligible, included in the study, completing follow-up, and analysed</td><td>Results -
- Lymphatic filariasis in Mauke</td></tr>
</table>

		- Malaria in the Garki Project, Nigeria<br>- Enteric pathogens in Haiti and the USA
13b	Give reasons for non-participation at each stage	NA
13c	Consider use of a flow diagram	NA
Descriptive data		
14a	Give characteristics of study participants (eg demographic, clinical, social) and information on exposures and potential confounders	Results -<br>- Lymphatic filariasis in Mauke<br>- Malaria in the Garki Project, Nigeria<br>- Enteric pathogens in Haiti and the USA
14b	Indicate number of participants with missing data for each variable of interest	NA
14c	Summarise follow-up time (eg, average and total amount)	NA
Outcome Data		
15	Report numbers of outcome events or summary measures over time	Fig 1, 2, 3, 4, 5
Main Results		
16a	Give unadjusted estimates and, if applicable, confounder-adjusted estimates and their precision (eg, 95% confidence interval). Make clear which confounders were adjusted for and why they were included	Fig 1, 2, 4, 5
16b	Report category boundaries when continuous variables were categorized	Methods<br>- Statistical methods<br>S1 Fig<br>S3 Fig
16c	If relevant, consider translating estimates of relative risk into absolute risk for a meaningful time period	NA
Other Analyses		
17	Report other analyses done—eg analyses of subgroups and interactions, and sensitivity analyses	S1, S2, S3, S4 Fig
**Discussion**		
Key Results		
18	Summarise key results with reference to study objectives	Discussion
Limitations		

19	Discuss limitations of the study, taking into account sources of potential bias or imprecision. Discuss both direction and magnitude of any potential bias	Discussion -<br>- Limitations and next steps
Interpretation		
20	Give a cautious overall interpretation of results considering objectives, limitations, multiplicity of analyses, results from similar studies, and other relevant evidence	Discussion -<br>- Interpretation
Generalisability		
21	Discuss the generalisability (external validity) of the study results	Discussion -<br>- Limitations and next steps
**Other Information**		
Funding		
22	Give the source of funding and the role of the funders for the present study and, if applicable, for the original study on which the present article is based	Financial Disclosure
