# Supplementary material for: Measuring changes in transmission of neglected tropical diseases, malaria, and enteric pathogens from quantitative antibody levels
Source: PLoS Negl Trop Dis. 2017 May 19;11(5):e0005616. doi: 10.1371/journal.pntd.0005616 (PMC5453600; doi:10.1371/journal.pntd.0005616)
Supplement: S1 Fig — a, W. bancrofti Wb123 age-dependent seroprevalence curves. b, mean Wb123 seroprevalence by age category. c, Kernel smoothed density distributions of Wb123 antibody levels in 1975 and 1992, along with a seropositivity cutoff value. (PDF) [file pntd.0005616.s004.pdf]

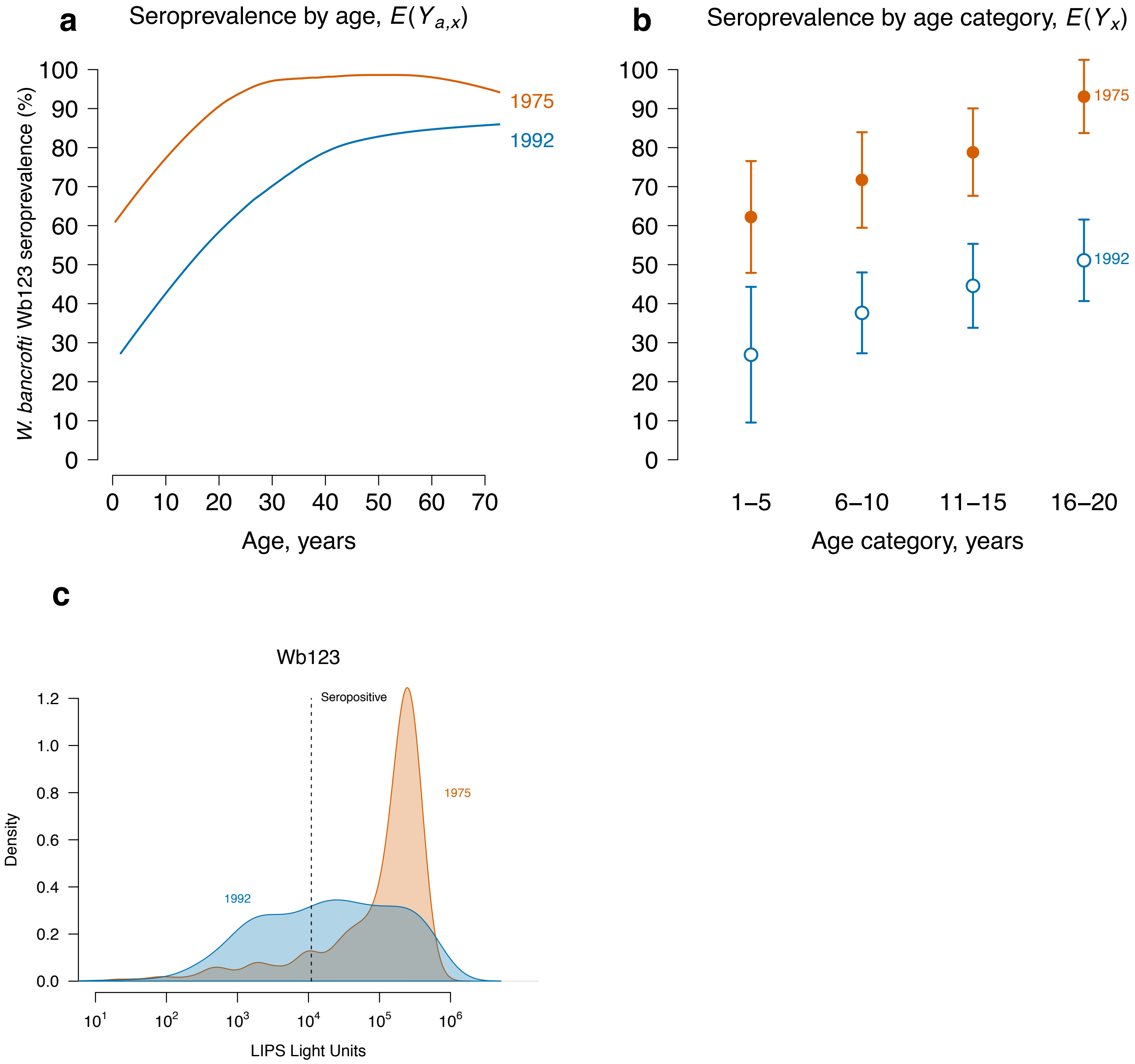

S1 Figure : A shift in the *Wuchereria bancrofti* Wb123 age-dependent seroprevalence curve measures a reduction in transmission due to mass drug administration (MDA) on Mauke Island. **a**, *W. bancrofti* Wb123 age-dependent seroprevalence curves. **b**, mean Wb123 seroprevalence by age category. Wb123 measured from blood specimens collected from residents in 1975 (N=362) before MDA and again in 1992 (N=553), five years following a single, island-wide MDA with diethylcarbamazine. **c**, Kernel smoothed density distributions of Wb123 antibody levels in 1975 and 1992, along with a seropositivity cutoff value (10968 light units) identified to maximize sensitivity and specificity using positive and negative controls (Kubofcik et al. PLOS Negl Trop Dis. 2012;6: e1930) and used in the seroprevalence analyses. The source data used to generate this figure are here: `https://osf.io/8tqu4` (mauke), and the scripts used to generate the figure are here: `https://osf.io/ek3sx` (mauke).
