# Supplementary material for: Measuring changes in transmission of neglected tropical diseases, malaria, and enteric pathogens from quantitative antibody levels
Source: PLoS Negl Trop Dis. 2017 May 19;11(5):e0005616. doi: 10.1371/journal.pntd.0005616 (PMC5453600; doi:10.1371/journal.pntd.0005616)
Supplement: S2 Fig — (PDF) [file pntd.0005616.s005.pdf]

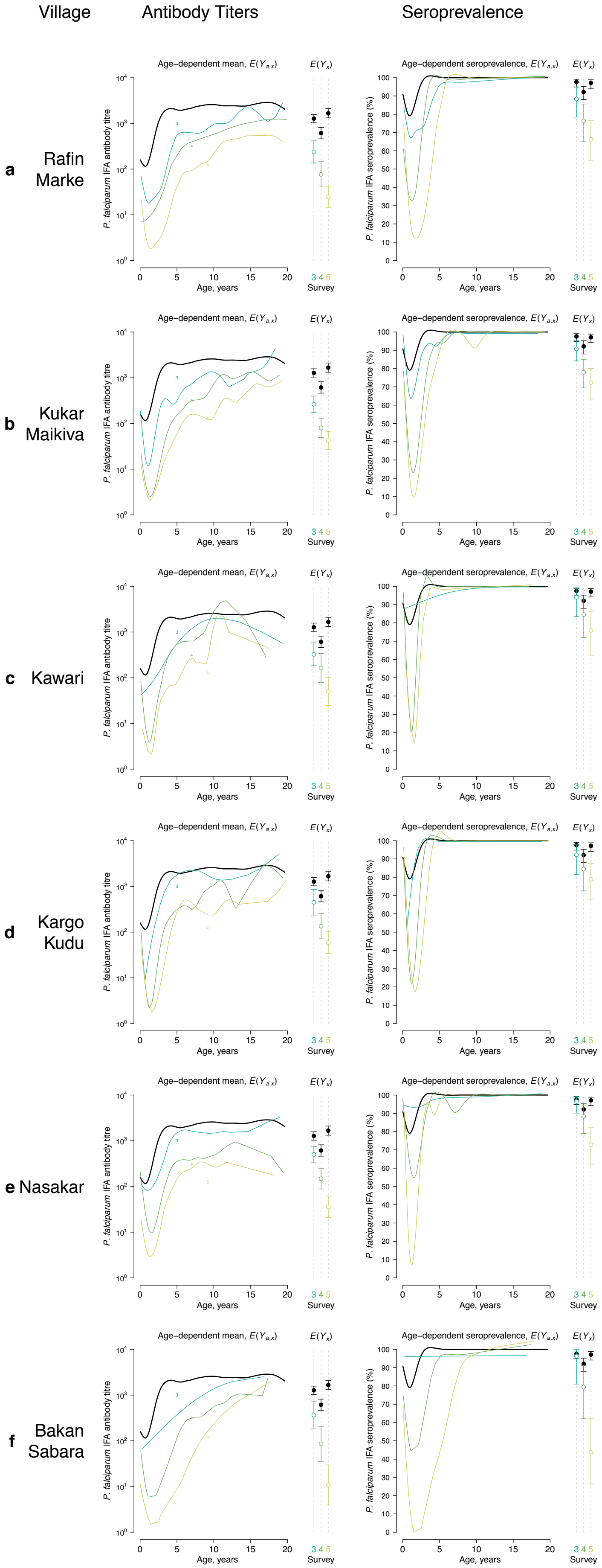

S2 Figure : Caption on the following page.

S2 Figure: *Plasmodium falciparum* age-antibody curves estimated separately for each intervention village (**a-f**) measure reductions in malaria transmission due to intervention in the Garki Project, Nigeria (1970-1976). Antibody response measured with the indirect fluorescent antibody (IFA) test for *P. falciparum* during the active intervention period (survey rounds 3-5, at 20, 50, and 70 weeks following the start of intervention). The left column includes curves and means using quantitative antibody titers and the right column includes estimates using seroprevalence. Each curve and mean was estimated with between 19 and 119 observations, depending on the village and survey round. Error bars show 95% confidence intervals for the summary means. Each village panel includes the same curve for the two control villages (black) for comparison. Control measurements were combined across survey rounds within each period when plotting the curves to facilitate visual comparison of shifts in transmission between surveys. The source data used to generate this figure are here: `https://osf.io/8tqu4` (garki), and the scripts used to generate the figure are here: `https://osf.io/ek3sx` (garki).
