# Supplementary material for: Measuring changes in transmission of neglected tropical diseases, malaria, and enteric pathogens from quantitative antibody levels
Source: PLoS Negl Trop Dis. 2017 May 19;11(5):e0005616. doi: 10.1371/journal.pntd.0005616 (PMC5453600; doi:10.1371/journal.pntd.0005616)
Supplement: S3 Fig — a, Cryptosporidium parvum recombinant 17-kDa antigen; b, Cryptosporidium parvum recombinant 27-kDa antigen; c, Giardia intestinalis variant-specific surface protein-5 (VSP-5); d, Entamoeba histolytica lectin adhesion molecule (LecA); e, enterotoxigenic Escherichia coli (ETEC) heat labile toxin subunit. f, Salmonella spp. lipopolysaccharide (LPS) Group B; g, Norovirus Group I.4; h, Norovirus Group II.4 New Orleans. (PDF) [file pntd.0005616.s006.pdf]

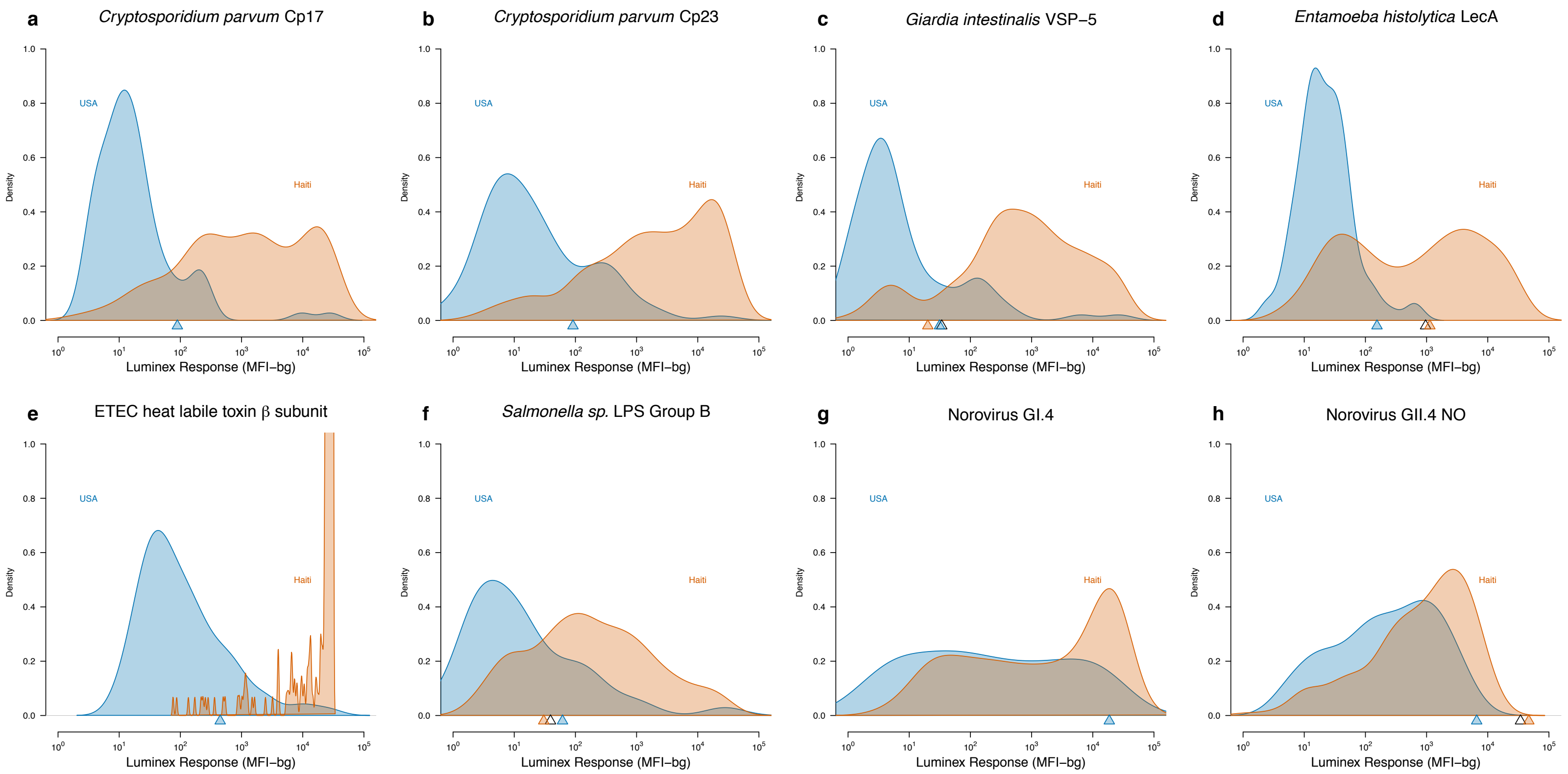

S3 Figure : Kernel density smoothed distributions of enteric antibody response in children < 5.5 years old in the United States (USA) and Haiti. Antibody response measured in multiplex on the Luminex platform using median fluorescence intensity minus background (MFI-bg). Triangles below the distributions indicate seropositivity cutoffs determined using finite gaussian mixture models fit to each country's measurements (filled triangles) or the combined sample set (open triangle), where cutoffs were determined using the mean+3*SD of the first gaussian component. Cutoff values beyond the range of observed measurements are not shown. **a.** *Cryptosporidium parvum* recombinant 17-kDa antigen; **b.** *Cryptosporidium parvum* recombinant 27-kDa antigen; **c.** *Giardia intestinalis* variant-specific surface protein-5 (VSP-5); **d.** *Entamoeba histolytica* lectin adhesion molecule (LecA); **e.** enterotoxigenic *Escherichia coli* (ETEC) heat labile toxin β subunit. Note: the Y-axis is truncated at 1.0 but extends to 10.0 for this antibody; **f.** *Salmonella spp.* lipopolysaccharide (LPS) Group B; **g.** Norovirus Group I.4; **h.** Norovirus Group II.4 New Orleans. The source data used to generate this figure are here: https://osf.io/8tqu4 (enterics), and the scripts used to generate the figure are here: https://osf.io/ek3sx (enterics).
