# Supplementary material for: Measuring changes in transmission of neglected tropical diseases, malaria, and enteric pathogens from quantitative antibody levels
Source: PLoS Negl Trop Dis. 2017 May 19;11(5):e0005616. doi: 10.1371/journal.pntd.0005616 (PMC5453600; doi:10.1371/journal.pntd.0005616)
Supplement: S4 Fig — a, Cross-validated estimates of R2 for the super learner ensemble and its constituent models/algorithms across example populations and pathogens. b Super learner ensemble estimates of age-dependent antibody curves for different populations and pathogens including the full library as well as a restricted library that excluded two highly adaptive algorithms (Random Forest and MARS). (PDF) [file pntd.0005616.s007.pdf]

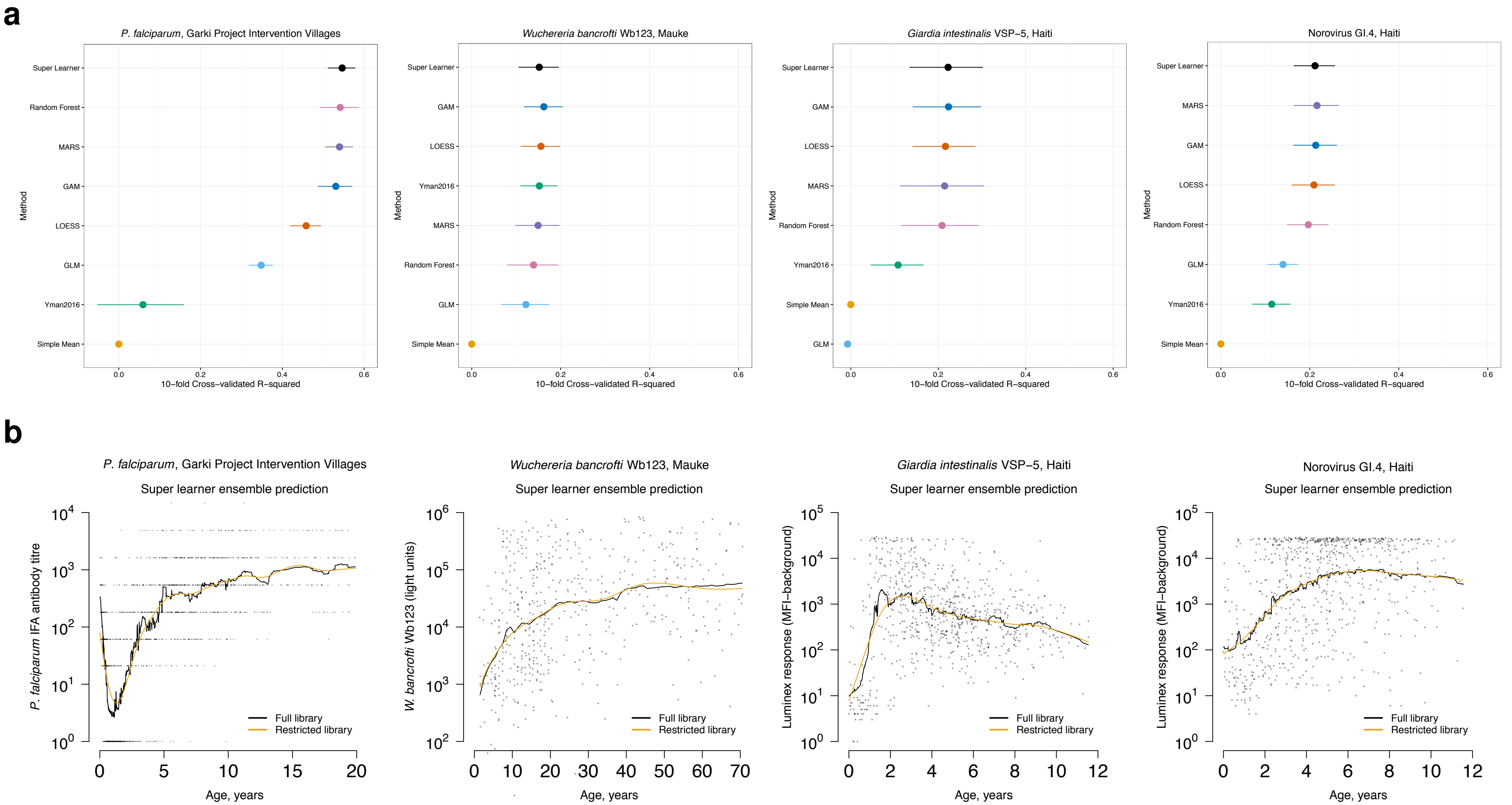

S4 Figure : Performance of the ensemble and individual models/algorithms. The super learner algorithm combines predictions from an ensemble by selecting a weighted combination of individual model predictions using cross-validation to optimize the bias-variance tradeoff – in this case, mean squared error (MSE). We estimated the cross-validated MSE and the related cross-validated R^2 of the super learner along with each of its constituent models/algorithms across a range of populations and pathogens in the analysis. Cross-validated R^2 represents the percentage of outcome variability explained beyond estimating the simple mean. **a**, Cross-validated estimates of R^2 for the super learner ensemble and its constituent models/algorithms across example populations and pathogens. Horizontal lines represent twice the standard error of the R^2 estimates measured across 10 cross-validation splits. In all cases, the algorithms only included age as a feature in antibody level prediction. In the Garki Project, where additional information was available, including additional covariates (sex, wet vs. dry season, village membership) did not markedly improve R^2 for any algorithm or the ensemble. **b** Super learner ensemble estimates of age-dependent antibody curves for different populations and pathogens including the full library (all members listed in **a**) as well as a restricted library that excluded two highly adaptive algorithms (Random Forest and MARS).The most highly adaptive algorithms that we considered (random forest and MARS) often led to jagged age-antibody curves, and excluding them led to consistent but smoother curves. We therefore used the restricted library in other analyses. Abbreviations: GAM: generalized additive models with natural splines; GLM: generalized linear model; LOESS: Locally weighted regression; MARS: Multivariate adaptive regression splines; Yman2016: Antibody acquisition model proposed by Yman et al. [Sci Rep 2016; 6:19472]. The source data used to generate this figure are here: `https://osf.io/8tqu4`, and the scripts used to generate the figure are here: `https://osf.io/ek3sx`.
